# A Novel Subset of Triple-Negative Breast Cancers with Unique Histology and Immunohistochemical Expression

**DOI:** 10.30699/IJP.2022.139734.2526

**Published:** 2022-03-08

**Authors:** Swaminathan Kalyanasundaram, Shantaraman Kalyanaraman, Hidhaya Kaleelullah Fathima, Vidhya Mohan, Kavitha Selvaraj

**Affiliations:** Tirunelveli Medical College, Tamilnadu, India

**Keywords:** Androgen receptor, Breast cancer, CK 5/6, EGFR, E- Cadherin, Triple-negative

## Abstract

**Background & Objective:**

Triple-Negative Breast Carcinoma (TNBC) is characterized by an absence of estrogen receptor, progesterone receptor and HER2 neu expression, with distinct molecular, histological and clinical features, aggressive clinical course and a poor prognosis. The objective was to evaluate the expression of Cytokeratin5/6 (CK 5/6), Epidermal Growth Factor Receptor 1 (EGFR 1), E-cadherin and Androgen receptor in tissue sections of TNBC.

**Methods:**

All modified radical mastectomy samples received negative for the three markers were subjected to further studies with CK5/6, EGFR 1, E- cadherin and Androgen receptor staining. The clinical and pathological data were tabulated and statistically analysed using the Chi-square test, and cross-tabulation was done to assess the correlation between these markers.

**Results:**

Of 94 samples classified as TNBC, 31 (33%) were positive for CK 5/6, 47 (50%) for EGFR, 32 (34%) for E Cadherin and Androgen receptor, respectively. We had one positive patient for all four markers, 13 patients were negative for all four. Thirty-five cases were positive for only one marker, 32 were positive for two markers, and 13 were positive for three markers. Analysis revealed certain interesting patterns, namely - E cadherin was the most common isolated marker expressed in our cohort of TNBC with 15 of 35 positives.

**Conclusion:**

This study highlights the presence of a unique subtype of TNBC, which are negative for all the four markers studied here, with unique histomorphology of absent tumour necrosis and stromal lymphocytic infiltration being unique.

## Introduction

Triple-Negative Breast Carcinoma (TNBC) is a subtype of breast cancer, devoid of ER, PR and Her2 expression, with minimal response to anti-HER2 drugs, shorter disease-free and overall survival (1-4) and a unique gene expression, with varied expressions of two basal-like proteins BL1 & BL2, an immunomodulatory, mesenchymal, mesenchymal stem cell-like, and luminal androgen receptor, each being associated with a different outcome (5, 6). TNBCs' are aggressive tumours with poor prognosis and have been reported to express High molecular weight cytokeratin (CK5/6, CK14 & CK17), epidermal growth factor receptor (EGFR), E-cadherin, and Androgen receptors (AR). High molecular weight cytokeratin (CK5/6, CK14 &CK17), and Epidermal growth factor receptor (EGFR), a member of the C-erb B family of tyrosine kinase receptor proteins, are postulated to be an effective therapeutic target(7-9),while E-cadherin, a transmembrane glycoprotein and hallmark of epithelial-to-mesenchymal transition, is mapped to the CDH1 gene located in the Ch16q22.1 locus, the inactivation of which results in larger tumours, higher tumour grades, greater risk of metastasis, and incidence of chemoresistance (10-14). Androgen receptors (AR) are promising drug targets. When detected in metaplastic apocrine cells and epithelial cells of the terminal duct lobular unit, it is factored as a favourable prognosis with a less aggressive clinical course and lesser chance of recurrence (15, 16). This study describes a unique pattern of expression of these protein moieties in our patients with TNBC.

## Material and Methods

The Hospital Cancer Registry data for 3 years since March 2016, had 102 patients with triple-negative breast carcinoma from among patients with breast carcinomas reporting to this tertiary care hospital in South India, based on their staining characteristics for ER, PR and the HER-2neu. Of these 102 patients, 94 were selected based on the availability of tissue samples in the pathology archives for additional studies and availability of a full complement of data that included basic demographic data, presenting com-plaints and their durations, menstrual, marital and lactation history, family history of malignancies and data pertaining to clinical presentation, mammogram, FNA cytology and histological examination. The data collected also included details of the gross tumor morphology-location of a tumour, its measurements, including least distances from resection margins, statuses of nipple & areola, overlying skin, tumour margins, presence or absence of tumour necrosis, numbers, levels and sizes of lymph nodes.

All procedures performed in the current study were approved by Institutional Ethical Committee in accor-dance with the 1964 Helsinki declaration and its later amendments. Informed consent was obtained from all individual participants included in the study.

**Histology of the Tumour**

 The archived material was verified for their patient identity, and tissue sections of 3 – 5μm were stained with H&E stain, reported and classified based on the WHO classification and graded based on the Notting-ham modification of Scarff – Bloom – Richardson system (Elston CW, Ellis IO; 1991) (17). The H&E slides were perused for type and grade of tumour, peri-tumoural lymphocyte infiltrates, the histological status of the nipple, areola, overlying skin, residual breast, resection margins, presence or absence of in situ components, nuclear grade, lymphovascular perm-eation, perineural lymphatic invasion, stromal reaction, assessment of stromal elastosis, microscopic involve-ment of lymph nodes. 

Peri-tumoural lymphocytes were classified based on the quantum of lymphocytes in the tumour substance as per criteria as mild (less than a 1/3^rd^ of the tumour shows lymphocytic infiltrates), moderate (1/3^rd^ to 2/3^rd^ of the tumour shows lymphocyte infiltrates) and marked (more than 2/3^rd^ of the tumour shows lymphocytic infiltrates) respectively. 

**Immunostaining of Tumor**

Primary rabbit monoclonal antibodies, a secondary antibody of Polyexcel Horse Radish Peroxidase Polymer (HRPP) and a colourimetric detection with Diamino-benzidine tetrachloride [DAB] were used.

**Fig. 1:**
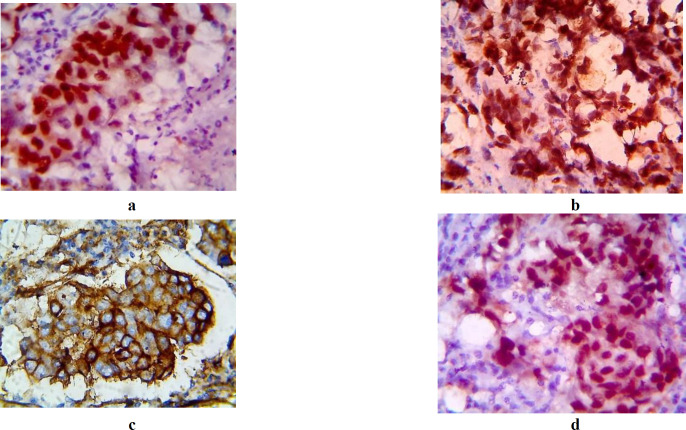
Figure 1a. Tumour cells showing cytoplasmic positive for CK5/6, [x400] Figure 1b. Tumour cells showing cytoplasmic and membranous positivity for EGFR1, [x400] Figure 1c. Tumour cells positivity for E Cadherin, [x400] Figure 1d. Tumour cells positivity for Androgen receptor, [x400]

1. **CK5&6: **(EP24&EP67clone- Biogenex, Fremont, CA) The positive immuno-staining CK5/6 was seen in the cytoplasm(Figure 1a)
2. **EGFR:** EGFR (EP22 (R) clone, Biogenex, Fremont, CA), EGFR positivity was observed in the cytoplasm and membrane of the tumour cells. (Figure 1b)
3. **E-Cadherin: **(NCH-38, Dako; Dilution 1:200). E-cadherin expression was semi-quantitatively analysed according to the percentage of cells showing membrane positivity: 0 (0 to 10%); 1+ (10 to 30%); 2+ (30 to 70%); 3+ (>70%). E-cadherin expression was considered positive if the score was ≥ 2, and negative if the score was ≥1 (Figure 1c).

**Androgen Receptor**

(F39.4.1 1:100 dilution, Biogenex, Fremont, CA) Expression of androgen receptor was analyzed as the percentage of cells showing positivity and prostate carcinoma cases were taken as a positive control. The cut-off value for AR positivity was set at >1% of tumour cell nuclei stained positive (Figure 1d)**.**

## Results

This study group was 25 to 84 years old with a mean age of 50.4 years, while 36.2% of the patients were between 50 and 60 years of age. The patient's had a mean age at menarche of 12.2 years. Of all the 94 patients included, all were married, of whom 8 were nulliparous, and the rest had children. All the women with children had lactated with periods of lactation ranging from 8 months to 18 months.

Of the 94 patients, 12 had a family history of cancers, 18 reported retraction of the nipple, 12 had skin ulceration, 6 had serous discharge from the nipple, and 2 had the peau-d'orange appearance of breast skin. The duration of clinical symptoms ranged between 1 and 20 months.

Fine-needle aspiration cytology (FNAC) was done in all of the cases.Eighty-five patients underwent Tru-cut biopsies to confirm the diagnosis of malignancy in 81 patients, 93 of the total patients underwent modified radical mastectomy with axillary clearance. In contrast, the remaining patient underwent a simple mastectomy.

The mastectomy specimens were received in the histopathology division of the department of patholo-gy, fixed in 10% neutral buffered formalin, dissected as per College of American Pathologists (18). The lesions were identified and measured, and the tumour size was classified into three groups. This study had 53 patients (56.3%) with tumour sizes of 2-5cm, 23 patients (24.4%) with tumour sizes of more than 5cm and 18 patients (19.1%) with a tumour size of less than 2cm. Invasive ductal carcinoma - NOS type is the most common histological type identified in 88 patients, followed by metaplastic carcinoma (3 patients) and atypical medullary carcinoma, medullary carcinoma and invasive lobular carcinoma. Grade II histology was seen in 67patients(71.3%), tumour necrosis was seen in 30 patients (31.9%) both on gross examination and microscopy, stromal lymphocytic infiltration was seen in 29 patients (30.9%) patients, stromal fibrosis in 65 patients (69.1%) and none of the patients showed stromal elastosis. Lymphovascular invasion was seen in 13 patients (13.8%), perineural lymphatic invasion in 8(8.5%) patients, infiltration of overlying skin was observed in 15 patients and infiltration of the nipple and areola in 9 patients. The surgical resection margins were free of tumours in 93 patients.

Among the 94 patients studied, CK 5/6 staining was seen in the cytoplasm of the tumour cells in 31 patients (33%), EGFR in 47 patients (50%), E-Cadherin in 32 patients (34%) and Androgen receptor in 32 patients (34%). One of the patients was positive for all the four IHC markers, and 13 patients (13.8%) were negative for all the four markers, while 35 (37.2%) were positive for only one, 32 (34.04%) for two and 13 (13.8%) for three of the four IHC markers studied (Table 1).

Of the 13 patients who were negative for all the four IHC markers studied, tumour necrosis and stromal lymphocyte infiltration were absent in all 13 patients. The mean age of these patients was 46.9 years, marginally lower than the mean age of the study population of patients with TNBC at 50.4 years.

**Table 1:** Distribution of positivity with various immunohistochemical markers in the triple negative breast cancer

S No	Content	Total	Grade 1	Grade 2	Grade 3
1	Total number of cases	94	04	67	**23**
2.	Mean age (yrs)	50.4	40.7	49.8	**63.8**
3.	Stromal Lymphocytic infiltration	29	01	17	**11**
4.	CK 5/6 Positivity	31	01	20	**10**
	EGFR 1 Positivity	47	01	28	**18**
	E Cadherin	32	00	24	**08**
	Androgen	32	03	24	**05**
5	Single Marker Positivity				
	CK 5/6 Positivity	00	00	00	00
	EGFR 1 Positivity	12	00	07	**05**
	E Cadherin	15	00	11	**04**
	Androgen	08	04	04	**00**
6	Double Marker Positivity	32	01	20	**11**
7	Triple Marker Positivity	13	00	09	**04**
8	Positive For 4 Markers	01	00	00	**01**
9	**Negative For All 4 Markers**	**13**	**00**	**13**	**00**

## Discussion

Triple-Negative Breast Cancers, forming 15% of the breast tumours (19), expresses characteristics of a basal-like subgroup of ductal cells, has a decreased expression of ER, PR and Her2,increased expression of proliferative markers (20), greater chances of late re-lapses (21), and molecular pathophysiology that remains poorly understood even today (22). Hence, TNBCs are considered a diagnosis of exclusion rather than a definite histological entity (23). But this subgroup of tumours remains a clinical challenge as they have lesser responses to endocrine or anti-HER2 drug regimens (24, 25).

Triple-negative breast cancers have been reported in women above 40 years of age with a mean age of 50 years (26, 27)as reported in this cohort of 94 patients with a mean age of 50.4 years, while it is majorly differently reported that TNBCs occur in younger women of Asian Indian, black or Hispanic races(28, 29).

Most of the patients diagnosed with TNBC are reported to be in stage II with large tumour sizes attributed to the rapid growth rates of this subtype of a tumour with mean tumour sizes of 2.78±0.012 cms (29, 30). Most of our patients (56.3%) had a maximum tumour size of 2-5 cms.

Histologically, classical TNBC is best identified by IHC staining, while non-classical rare forms of TNBC have to be described, e.g. adenoid-cystic carcinomas-(31-34), low-grade adenosquamous carcinoma (35), fibromatosis-like metaplastic carcinoma (36), and secretory carcinoma (37) and it is also reported that these rare subtypes have a better prognosis, and are low-proliferating tumours. The classical TNBC is mostly of invasive ductal carcinoma (NOS) type. TNBCs have also been associated with a DCIS component in 45-50% of the patients (38-41), which was not replicated in this study. The in-situ component was seen in only 3.2% of patients. The presence of tumour necrosis has been documented in various studies with 74% to 58.3% of TNBC patients (42-44), while our cohort had tumour necrosis in only 31.9% of patients. Stromal lymphocytic infiltration was docum-ented in 49%-56% of patients with TNBC (41, 42), while our cohort reported only 30.9%, which is considerably lower. In this study, the lymphovascular invasion was identified in 13.8% of patients, similar to experiences reported in 15-18% of the patients (40, 45). Most of our patients were Grade II (71.3%), while Nassar *et al.* (2010) reported that 77%of patients were high grade (46).

Basal Cytokeratins (CK 5, 14, and 17) in Breast Cancer are markers of aggressive clinical behaviour (47, 48). An interesting observation was that in CK positive TNBC, tumour necrosis and infiltrating borders were common findings. At the same time, lymphocytic infiltration and prominent nucleoli were less common, and no significant association was identified between CK 5/6 and morphologic features in TNBC (44). It has also been observed that 74% and 67.7% TNBC patients showed expression of either or both of CK 5/6 and EGFR, as we have also reported here (49, 50). CK 5/6 and EGFR positivity was predominantly associated with elderly TNBC patients (>60 years of age) similar to an observation of Tan *et al.* (2009) (27) maximum tumour size ranging from 4-5cm similar to an observation of Thike *et al.* (2010) (51), grade 3 tumours at 45.16% which was lesser than the observations of Thike *et al.* (2010) (51) at 77%, Hashmi *et al. *(2014) (52) at 63.4% and Rao *et al. *(2013) (49) at 76 %. Of the total 13 patients who had lymphovascular permeation, CK 5/6 and EGFR were positive in 69.2% and 76.9%, respectively and those with stromal lymphocytic infiltration, CK 5/6 and EGFR were positive in 65.5%

E-Cadherin positivity was equally distributed among the age groups 51 to 60 years (38.2%) and 40 to 50 years (39.3%), similar to the report of Rakha *et al.* (2007) (30). The expression of E-Cadherin was seen associated with tumour sizes less than 5 cm (26 of 32 patients), emphasizing that as the size of the tumour mass increased, the expression of E-cadherin was reduced, sharing a conclusion of Tang *et al.*(2012) (53)

Similarly, E-cadherin's expression in Grade III carcinoma was only 9.4% explaining the hypothesis that there was a downregulation of expression of the E-Cadherin with increasing grades of the tumour (54). E-Cadherin was expressed well with the absence of tumour necrosis (70%) and less expressed with stromal lymphocytic infiltration (41.40%), reinforcing a similar report (55). Our study shows a significant correlation between lack of E-cadherin expression with the tumour size, histological grade of the tumour, tumour necrosis and axillary nodal status reflecting the progression of malignancy.

Androgen receptor (AR), a steroid receptor linked to transcription factor involved in cell proliferation and apoptosis, was seen in 34% of the TNBC patients in our study, which correlates with other experiences, of which one author reported 2,000 invasive breast cancers, where in AR was positive in only 32% of the TNBCs.

Expression of Androgen receptor was more among women with TNBC in the age range of 51 to 60 years at 35.3%, among patients having tumour sizes<5-cm confirming that as tumour mass increased, the expression of androgen receptor decreased. A decreased expression of AR was seen in grade 3 tumours, with the presence of tumour necrosis, and was negative in 84.6% of patients with lymphocytic infiltration. Sutton *et al.* affirm the above factors, and those AR-positive TNBCs have a lesser chance of metastasis. Our study showed a significant correlation between expression of androgen receptors with the subject's age, tumour size, histological grade of the tumour and axillary nodal status. 

Analysis of the immune profile revealed certain interesting patterns, namely – E-cadherin was the most common isolated marker expressed in our cohort of TNBC with 15 of 35 positives. Isolated positive staining was not seen with CK 5/6, which Chandrika Rao et al. (2013) (49) affirmed. Isolated positivity for androgen receptors was seen in 8 patients. The most common double markers positive together was CK 5/6 and EGFR seen in 13 patients. Of the 13 patients with triple positives, all were of histological grade II and III.

There was a unique subset of TNBC, negative for all four markers. Tumour necrosis and stromal lympho-cytic infiltration were absent in all these cases. This unique subset needs further molecular studies to characterize. The limitation of this study is that the sample size for the unique subset is limited and more samples may provide greater insight into the unique subtype of breast carcinoma. 

## Conclusion

TNBC, a heterogeneous group of tumours, possess distinctive pathological features and are an aggressive subtype with a poor prognosis. This study reaffirms the utility of IHC markers in characterizing TNBCs, to stratify the patients into favourable or unfavourable subtypes. A large subset of TNBC express EGFR can be a potential target for newer treatment modalities. This study highlights the presence of a unique subtype of TNBC, which is negative for all the four markers studied here, with a unique histomorphology of absent tumour necrosis and stromal lymphocytic infiltration. This subset needs to be characterized.

## Conflict of Interest

The authors declared no conflict of interest.

## Funding

None.
